# A New Insight into the Morphology of the Human Liver: A Cadaveric Study

**DOI:** 10.5402/2013/689564

**Published:** 2013-12-24

**Authors:** Sunitha Vinnakota, Neelee Jayasree

**Affiliations:** ^1^Maharajah's Institute of Medical Sciences, Nellimarla, Vizianagaram District, Andhra Pradesh 535217, India; ^2^Narayana Medical College, Chintareddy Palem, Nellore, Andhra Pradesh 524003, India

## Abstract

*Background*. Day to day advances in the fields of radiology like sonography and CT need to revive interest in the cadaveric study of morphological features of liver, as the accessory fissures are a potential source of diagnostic errors. Accessory fissures vary from single to multiple over different parts of the liver. *Aim*. In the present study the morphological features of human liver specimens were evaluated by macroscopic examination and morphometric analysis. *Methods*. The study was conducted on 58 specimens obtained from cadavers utilized for routine dissection for medical undergraduates from the year 2004 to 2012 in the Anatomy Department of MIMS Medical College. *Results*. In the present study the livers as described in the established anatomical literature with normal surfaces, fissures, and borders were considered normal. Out of the 58 specimens, 24 were normal without any accessory fissures or lobes and with normal contours. Two specimens were with hypoplastic left lobes. Lingular process of left lobe was observed in only one specimen. *Conclusions*. Knowledge of the various accessory fissures of liver prevents misdiagnosis of cystic lesions or any pathological lesions of the liver.

## 1. Introduction

**Figure 1:**
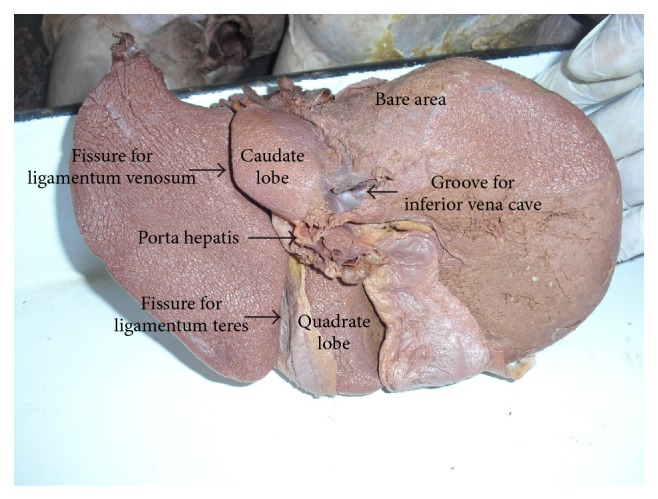
Shows the posterior and inferior surfaces of normal liver.

Liver is the most massive of the viscera, occupying a substantial portion of abdominal cavity, that is, right hypochondrium and epigastrium, and extending into left hypochondrium as far as left lateral line [1]. It is a wedge shaped organ with its narrow end pointing towards left. It is convex in the front, to the right, above, and behind, and is somewhat concave inferiorly, where it is moulded to the shapes of the adjacent viscera [2]. Even though the surface is smoothly continuous, liver is customarily apportioned by anatomists into a larger right and a much smaller left lobe by the line of attachment of the falciform ligament anteriorly and the fissure for ligamentum teres and ligamentum venosum on inferior surface. In addition to the right and left lobes, there are two additional lobes, a quadrate lobe in the front and the caudate lobe behind, separated from each other by the porta hepatis (Figure 1).

Quadrate lobe visible on the inferior surface appears somewhat rectangular and is bounded in the front by the inferior border, on the left by fissure for ligamentum teres, behind by porta hepatis, and on the right by the fossa for the gall bladder (Figure 1).

The caudate lobe is visible on the posterior surface, bounded on the left by fissure for ligamentum venosum, below by porta hepatis, and on the right by the groove for inferior vena cava. Above, it continues into the superior surface. Below and to the right, it has a narrow caudate process. Below and to left it has a small round papillary process (Figure 1).

Gross abnormalities of liver are rare despite its complex development. The more common gross abnormalities are irregularities in form, number of lobules, and in the presence of cysts. A less common abnormality is occurrence of one or more accessory liver or lobes [3].

## 2. Materials and Methods

58 liver specimens available in the Anatomy Department of Maharajah's Institute of Medical Sciences constituted the study material. The liver specimens had been removed from adult human cadavers during routine dissection for medical undergraduate students from the year 2004 to 2012 and then preserved in 10% of formalin.

Each lobe of the liver, that is, right lobe, left lobe, caudate lobe, and quadrate lobe was studied in detail for the size, shape, accessory fissures, and accessory lobes. The morphometric data included weight, maximum vertical height, and maximum width.

## 3. Results

### 3.1. Morphometric Data

The weight of the livers ranged from 900 grams to 2 kg. The maximum height ranged from 9 cm to 24 cm. The maximum width ranged from 11.8 cm to 20 cm.

### 3.2. Morphological Aspects

In the present study the livers with normal surfaces, fissures, and borders were considered normal. Out of the 58 specimens, 24 (41.37%) were normal without any accessory fissures or lobes and with normal contour (Figure 1). Out of the remaining 34 specimens, 31 (53.44%) specimens, even though they appear normal, they had accessory fissures on the left lobe, right lobe, caudate lobe, and quadrate lobe, which resulted in the formation of accessory lobes. Hypoplastic left lobes were noted in 2 (3.44%) specimens. Lingular process of left lobe was present only in 1 specimen (1.72%) (Table 1).

#### 3.2.1. Right Lobe

**Figure 2:**
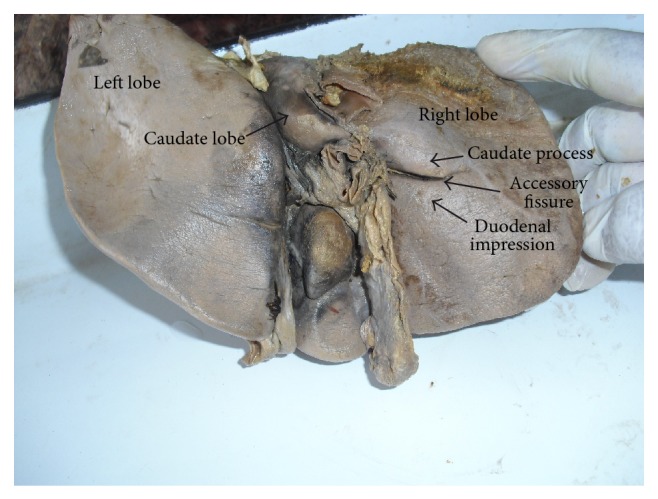
Shows accessory fissure between caudate process and duodenal impression on the inferior surface of right lobe of liver.

**Figure 3:**
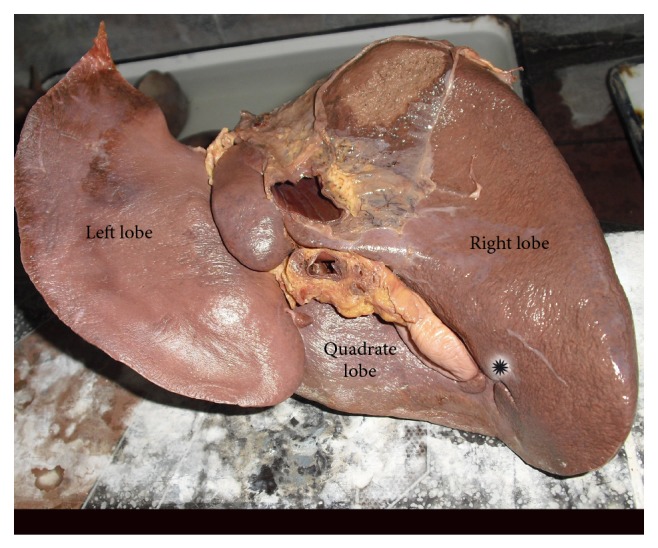
Shows accessory lobes present close to the base of gall bladder near the inferior border on inferior surface of right lobe of liver.

**Figure 4:**
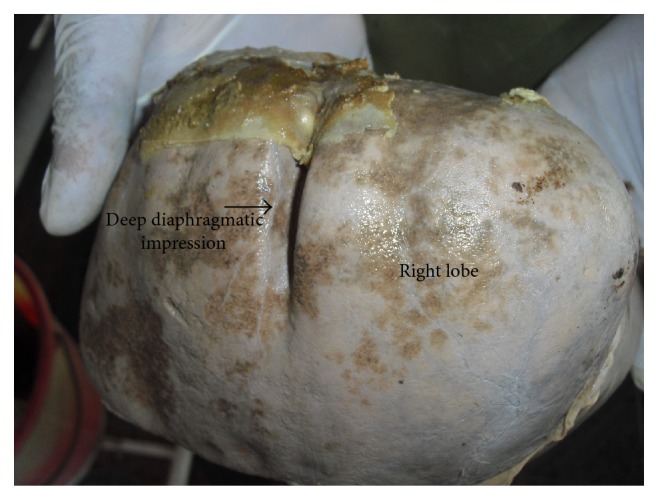
Shows diaphragmatic impressions on superior surface of right lobe of liver.

In 8 specimens, accessory fissures were seen in different areas of the right lobe (Table 2). Out of these 8 specimens, 6 specimens showed accessory fissures between caudate process and duodenal impression (Figure 2). These fissures varied in size and depth. In 1 specimen, 2 small accessory fissures resulted in 2 small accessory lobes present close to the base of gall bladder near the inferior border (Figure 3). One specimen with diaphragmatic impressions, that is, Netter type 7 [4] liver, was also noted in the present study (Figure 4).

#### 3.2.2. Left Lobe

**Figure 5:**
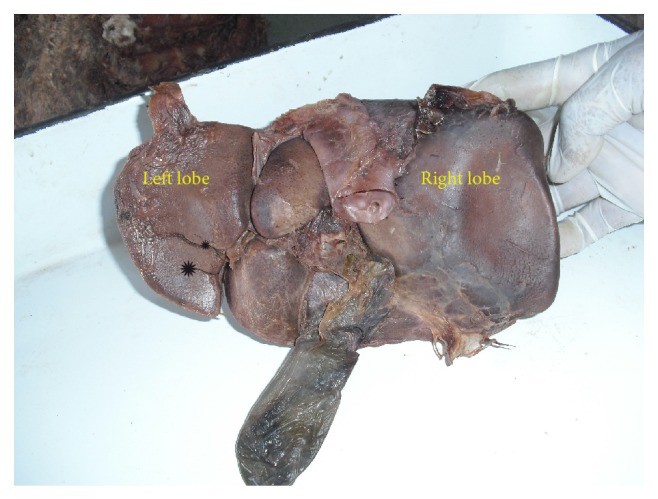
Shows accessory fissures that were noted over various areas of the left lobe of liver.

**Figure 6:**
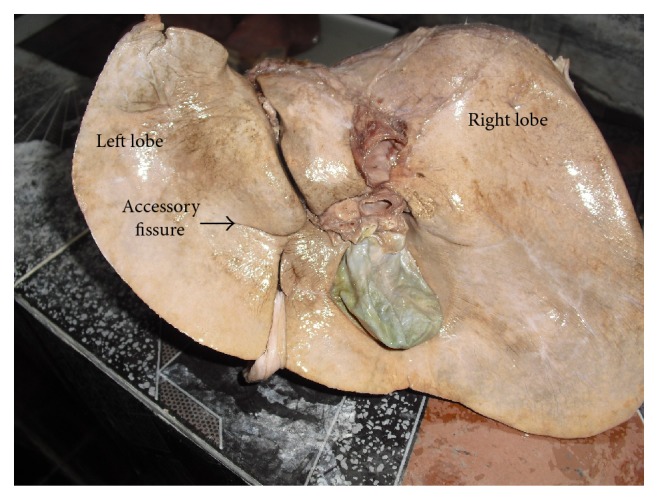
Shows accessory fissures that were noted over various areas of the left lobe of liver.

**Figure 7:**
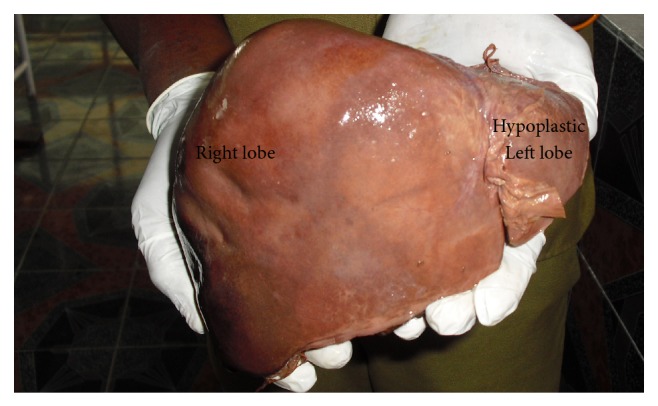
shows hypoplastic left lobe of liver.

**Figure 8:**
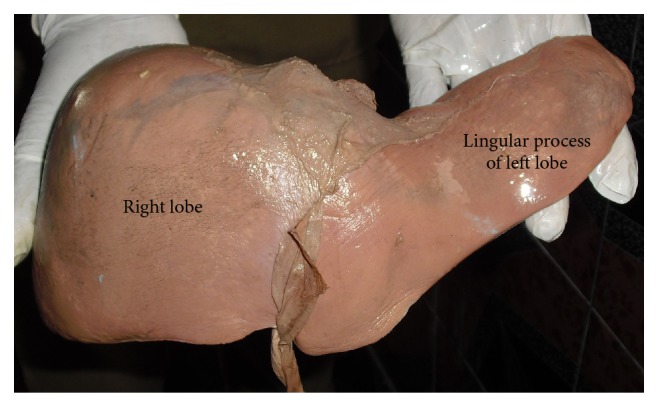
Shows lingular process of left lobe, that is, Netter type 5 liver.

In 6 specimens, accessory fissures were noted over various areas of left lobe of liver (Figures 5 and 6) (Table 2). Two specimens were with hypoplastic left lobes (Figure 7). In 1 specimen, lingular process of left lobe, that is, Netter type 5 [4], (Figure 8) was identified.

#### 3.2.3. Caudate Lobe

**Figure 9:**
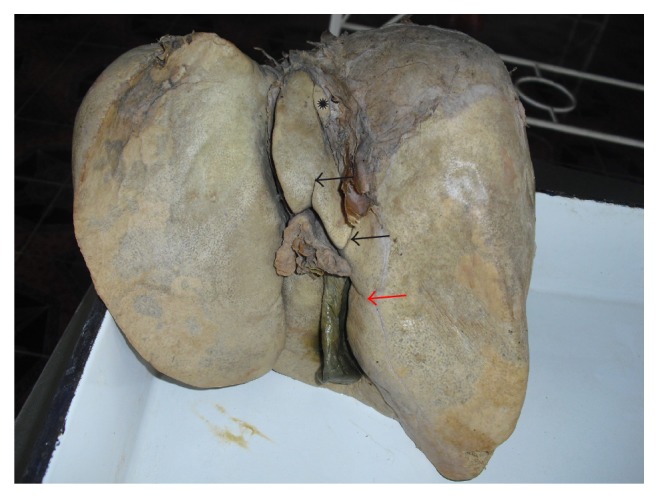
Shows accessory fissures and accessory lobes in caudate lobe of liver.

**Figure 10:**
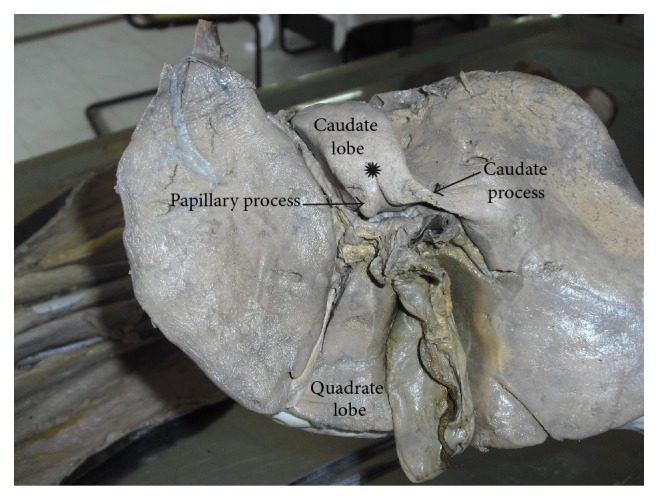
Shows accessory fissure between caudate process and papillary process of caudate lobe.

Out of 31, 8 specimens showed accessory fissures and accessory lobes in the caudate lobe (Table 2) (Figure 9). In 1 specimen the fissure was found to be between the caudate process and papillary process (Figure 10).

#### 3.2.4. Quadrate Lobe

**Figure 11:**
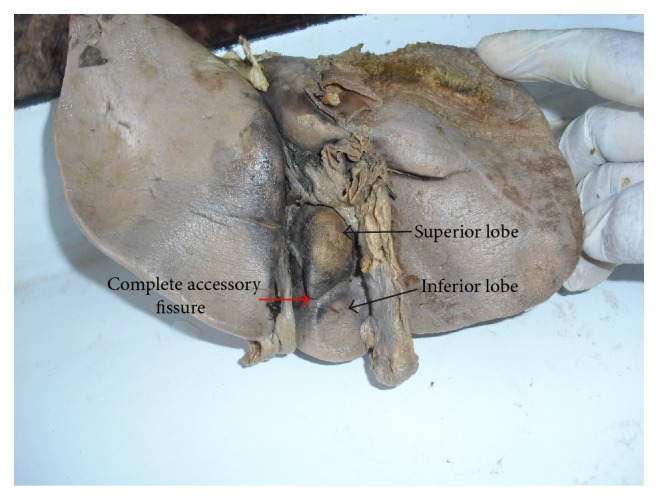
Shows complete transverse fissure dividing quadrate lobe into superior and inferior lobes.

**Figure 12:**
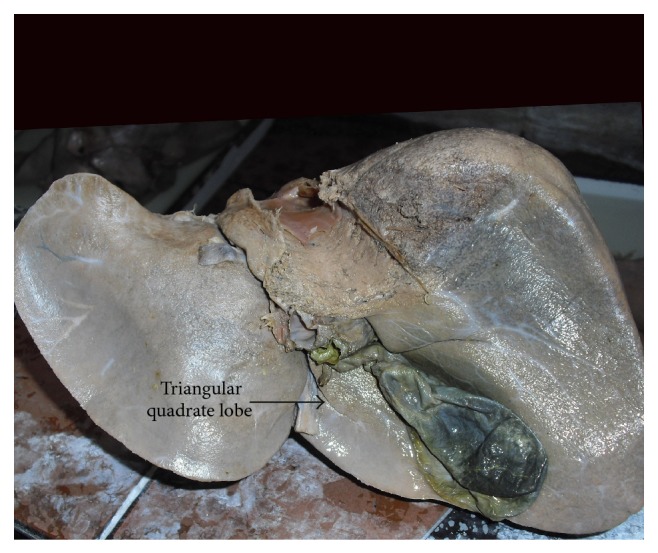
Shows triangular quadrate lobe of liver.

**Figure 13:**
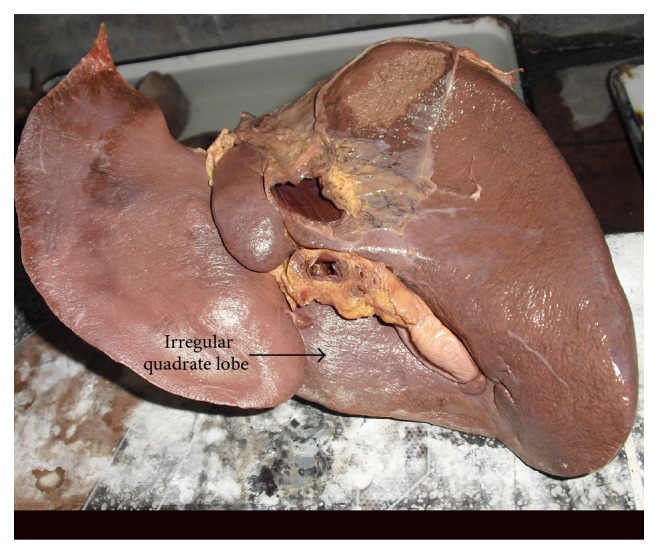
Shows irregular quadrate lobe of liver.

**Figure 14:**
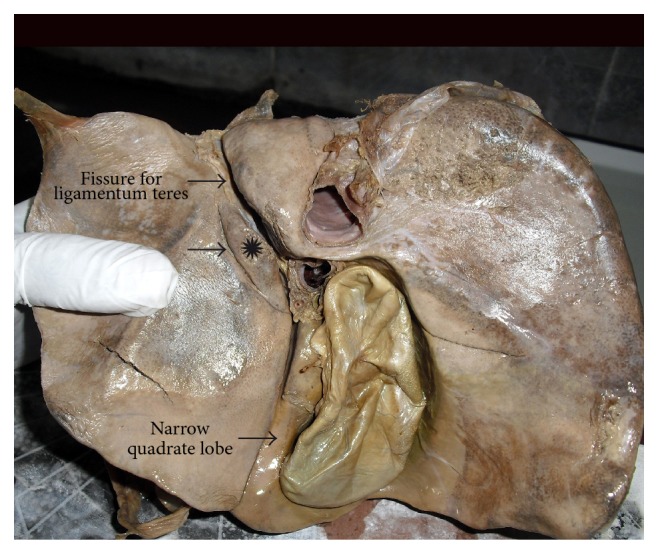
Shows very narrow quadrate lobe of liver.

**Figure 15:**
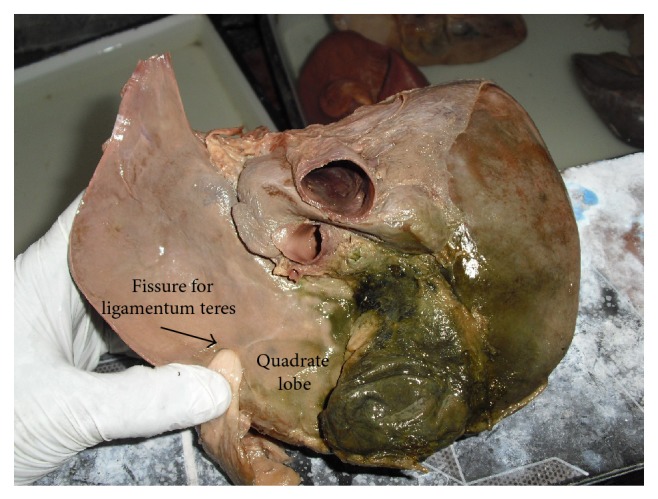
Shows quadrate lobe continuous with left lobe due to incomplete fissure for ligamentum teres.

Out of 9 specimens with accessory fissures in quadrate lobe, 1 specimen shows a complete transverse fissure dividing into a superior and an inferior lobe (Figure 11). Quadrate lobe varies in shape from triangular (Figure 12) to irregular (Figure 13) and it also varies in size from very narrow (Figure 14) to ill-defined and also continuous with left lobe due to the presence of an incomplete fissure for ligamentum teres (Figure 15).

**Figure 16:**
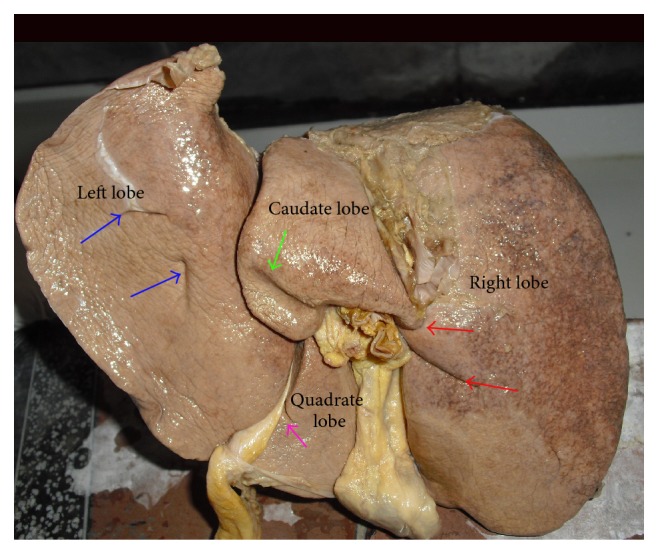
Shows accessory fissures over right, left, caudate, and quadrate lobes of liver.

**Figure 17:**
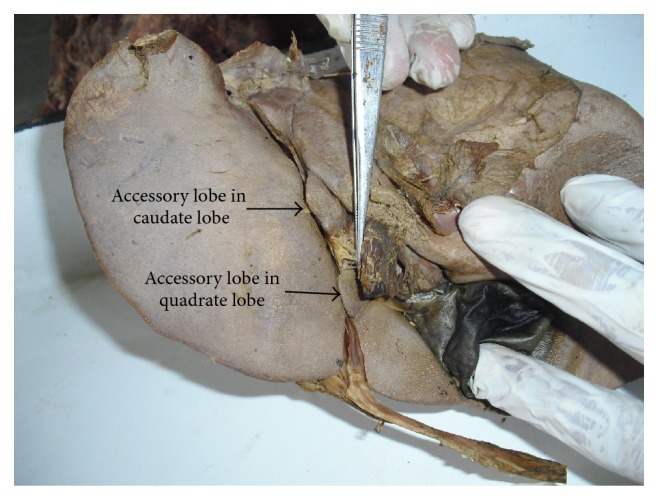
Shows accessory lobes present in both caudate and quadrate lobes.

In 1 specimen accessory fissures are present over the right, left, caudate, and the quadrate lobes (Figure 16). Accessory lobes are present in both the caudate and quadrate lobes in one specimen (Figure 17).

## 4. Discussion

Of all the digestive organs, the liver is the one which starts its organogenesis early during 3rd week of intrauterine life and develops most rapidly [5]. Gross abnormalities of the liver are rare in spite of its complex development. The more common gross abnormalities are irregularities in form and less common abnormality is the occurrence of one or more accessory livers or lobes [3]. Bradley [6] has done much to elucidate the development of liver. The single liver in some lower animals like pig and dog has distinct lobules separated by strands of connective tissue and sometimes the human liver shows this variation by reversion [3]. The variations in the anatomy of human liver have been classified as congenital or acquired [7]. The congenital anomalies of liver can be divided into anomalies due to defective development and anomalies due to excessive development. Defective development of left lobe of liver can lead to gastric volvulus, whereas defective development of right lobe may remain latent or progress to portal hypertension [8]. The excessive development of liver results in the formation of accessory lobes of liver which may carry the risk of torsion [8]. Acquired changes in the liver morphology are represented by the following characteristic features: (1) linguiform lobes, (2) costal organ with very small left lobe, (3) deep renal impressions and “corset” type constriction and local inflammation of the organ or gallbladder [9]. The accessory hepatic fissures are potential sources of diagnostic errors on both sonography and CT (Y. H. Auh et al. [10]). Sonographically multiple accessory fissures also may be confused at first look with a macronodular liver, so a supplementary CT scan is often helpful for further evaluation [10]. Multiple hepatic fissures and lobes were more common on the under surface of liver, opposite to the quadrate lobe, and in the left lobe [11]. In the present study, 53.44% of livers are with accessory fissures over their various parts especially on the undersurface in accordance with Cullen [11]. In the present study 9 specimens have accessory fissures in quadrate lobe alone, whereas in 1 specimen, accessory fissures are present both in caudate and quadrate lobes (Figure 16) and in another liver all the lobes, that is, right, left, caudate, and quadrate lobes have accessory fissures on the under surface. So, in total 11 out of 31 specimens (35.48%) have accessory fissures in quadrate lobe.

## 5. Conclusion

There is a very sparse mention of the incidence of accessory fissures in various lobes of liver in the established anatomical literature. This study would certainly throw light on the importance of such variant appearances. Knowledge of accessory fissures over various parts of liver is important for radiologists, which prevents misdiagnosis of cystic lesions or any macroscopic pathological lesions of the liver.

## Figures and Tables

**Table 1:** Showing the incidence of normal and variant livers.

Morphological features	Number of specimens
Normal fissures and lobes	24 (41.37%)
Accessory fissures over different lobes	31 (53.44%)
Hypoplastic left lobe	2 (3.44%)
Lingular process of left lobe	1 (1.72%)

**Table 2:** Showing incidence of accessory fissures and accessory lobes in various lobes of liver.

Lobe	Accessory fissures	Accessory lobes
Right lobe	In 10 specimens	In 2 specimens
Left lobe	In 6 specimens	In 1 specimen
Caudate lobe	In 8 specimens	In 3 specimens
Quadrate lobe	In 9 specimens	In 4 specimens
